# Two Birds with One Stone: Hepatocellular Carcinoma and Small Cell Lung Cancer Imaged with [^18^F]Fluorocholine Positron Emission Tomography/Computed Tomography

**DOI:** 10.3390/diagnostics13162639

**Published:** 2023-08-10

**Authors:** Marco Cuzzocrea, Gaetano Paone, Giorgio Treglia

**Affiliations:** 1 Division of Nuclear Medicine, Imaging Institute of Southern Switzerland, Ente Ospedaliero Cantonale, 6500 Bellinzona, Switzerland; marco.cuzzocrea@eoc.ch (M.C.); gaetano.paone@eoc.ch (G.P.); 2 Faculty of Biomedical Sciences, Università della Svizzera Italiana (USI), 6900 Lugano, Switzerland; 3 Faculty of Biology and Medicine, University of Lausanne (UNIL), 1011 Lausanne, Switzerland

**Keywords:** PET, positron emission tomography, nuclear medicine, choline, hepatocellular carcinoma, small cell lung cancer

## Abstract

We describe the case of a 67-year-old male patient with a moderately differentiated hepatocellular carcinoma (HCC) of the right liver lobe who underwent [^18^F]fluorocholine positron emission tomography/computed tomography (PET/CT) for staging due to a suspicious lung lesion at previous CT scan. [^18^F]fluorocholine PET/CT showed increased radiopharmaceutical uptake in a liver lesion corresponding to the known HCC. Furthermore, a right pulmonary hilar lesion suspicious for metastatic spread of HCC showed increased radiopharmaceutical uptake. Surprisingly, the histological assessment of the thoracic lesion demonstrated the presence of small cell lung cancer (SCLC). The patient underwent treatment with radiation therapy and chemotherapy for the SCLC and selective internal radiation therapy (SIRT) for the HCC. The patient died after one year due to progressive SCLC. This case demonstrates that coexisting tumors showing increased cell membrane turnover, including SCLC, can be detected by [^18^F]fluorocholine PET/CT. In our case, [^18^F]fluorocholine PET/CT findings influenced the patient management in terms of histological verification and different treatment of the detected lesions.

**Figure 1:**
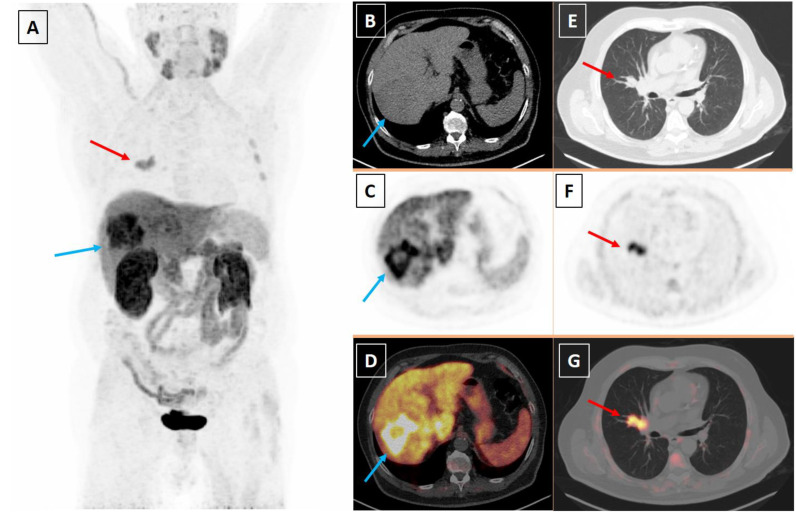
A 67-year-old male patient with a histological diagnosis of moderately differentiated hepatocellular carcinoma (HCC) and a suspicious right pulmonary lesion at computed tomography (CT) underwent [^18^F]fluorocholine positron emission tomography/computed tomography (PET/CT) for HCC staging. PET/CT was performed 30 min after the injection of 217 MBq of [^18^F]fluorocholine. Qualitative criteria for PET image analysis were used: areas of increased radiopharmaceutical uptake compared to the background were considered abnormal, excluding the sites of physiological radiopharmaceutical uptake. Furthermore, semi-quantitative PET image assessment using the maximal standardized uptake value (SUVmax) was performed. Maximum intensity projection PET image (**A**) and axial CT (**B**,**E**), PET (**C**,**F**) and fused PET/CT images (**D**,**G**) showed two areas of abnormal radiopharmaceutical uptake corresponding to the known HCC lesion in the right liver lobe (blue arrows) and to a right pulmonary hilar lesion (red arrows) suspicious for metastatic spread of HCC. SUVmax of liver and pulmonary lesions were 19 and 9, respectively. Surprisingly, the histological assessment of the thoracic lesion performed after [^18^F]fluorocholine PET/CT showed the presence of small cell lung cancer (SCLC). After multidisciplinary discussion, the patient underwent radiation therapy and chemotherapy for SCLC treatment and selective internal radiation therapy (SIRT) for HCC treatment. Unfortunately, the patient died one year after the [^18^F]fluorocholine PET/CT scan due to SCLC progression. According to evidence-based data, [^18^F]fluorocholine PET/CT is a useful imaging method for the staging and restaging of well differentiated and moderately differentiated HCC, which are tumors characterized by increased cell membrane turnover [1,2]. In particular, compared to conventional imaging techniques, [^18^F]fluorocholine PET/CT is very useful for detecting the metastatic spread of well differentiated and moderately differentiated HCC with several advantages compared to fluorine-18 fluorodeoxyglucose ([^18^F]FDG) PET/CT in terms of diagnostic performance [1,2]. [^18^F]fluorocholine PET/CT performed for oncological indications may incidentally detect benign or malignant lesions (second tumors) characterized by increased cell membrane turnover [3,4,5]. In particular, for lung cancers, it has been demonstrated that the uptake of [^18^F]fluorocholine is correlated with the proliferation of tumor cells; furthermore, the [^18^F]fluorocholine uptake mechanism is related to the overexpression of choline kinase and phosphorylcholine-cytidyl transferase in lung tumors [6]. A previous case reported the incidental detection of SCLC by [^18^F]fluorocholine PET/CT performed for a non-oncological indication (primary hyperparathyroidism) [7]. Our case demonstrated that coexisting tumors such as HCC and SCLC showing increased cell membrane turnover can be detected by radiolabeled choline PET/CT. In our case, radiolabeled choline PET/CT findings influenced the patient management in terms of histological verification and different treatment of the detected lesions.

## Data Availability

The data presented in this article are available on request from the corresponding author.
